# Limited effect of anti-rheumatic treatment on 15-prostaglandin dehydrogenase in rheumatoid arthritis synovial tissue

**DOI:** 10.1186/ar3851

**Published:** 2012-05-22

**Authors:** Karina Roxana Gheorghe, Syed Sadique, Patrick Leclerc, Helena Idborg, Ivonne Wobst, Anca Irinel Catrina, Per-Johan Jakobsson, Marina Korotkova

**Affiliations:** 1 Department of Medicine, Rheumatology Unit, Karolinska Institute/Karolinska University Hospital Solna, Stockholm, 171 76 Sweden; 2 Institute for Clinical Pharmacology, Johann Wolfgang Goethe-University Frankfurt, Theodor Stern Kai 7, 60590 Frankfurt/Main, Germany; 3 Actar AB, Nobels väg 3, Solna, 171 65 Sweden

## Abstract

**Introduction:**

Rheumatoid arthritis (RA) is a chronic inflammatory disease in which prostaglandin E_2 _(PGE_2_) displays an important pathogenic role. The enzymes involved in its synthesis are highly expressed in the inflamed synovium, while little is known about 15- prostaglandin dehydrogenase (15-PGDH) that metabolizes PGE_2_. Here we aimed to evaluate the localization of 15-PGDH in the synovial tissue of healthy individuals or patients with inflammatory arthritis and determine the influence of common RA therapy on its expression.

**Methods:**

Synovial tissue specimens from healthy individuals, psoriatic arthritis, ostheoarthritis and RA patients were immunohistochemically stained to describe the expression pattern of 15-PGDH. In addition, the degree of enzyme staining was evaluated by computer analysis on stained synovial biopsies from two groups of RA patients, before and after RA specific treatment with either intra-articular glucocorticoids or oral methotrexate therapy. Prostaglandins derived from the cyclooxygenase (COX) pathway were determined by liquid-chromatography mass spectrometry in supernatants from interleukin (IL) 1β-activated fibroblast-like synoviocytes (FLS) treated with methotrexate.

**Results:**

15-PGDH was present in healthy and inflamed synovial tissue, mainly in lining macrophages, fibroblasts and vessels. Intra-articular glucocorticoids showed a trend towards reduced 15-PGDH expression in RA synovium (p = 0.08) while methotrexate treatment left the PGE_2 _pathway unaltered both in biopsies *ex vivo *and in cultured FLS.

**Conclusions:**

Early methotrexate therapy has little influence on the expression of 15-PGDH and on any of the PGE_2 _synthesizing enzymes or COX-derived metabolites. Thus therapeutic strategies involving blocking induced PGE_2 _synthesis may find a rationale in additionally reducing local inflammatory mediators.

## Introduction

Rheumatoid arthritis (RA) is a chronic autoimmune disease characterized by inflammation and extensive proliferation within the joint synovial tissue and by recruitment and activation of immune cells and subsequent cartilage and bone destruction. Rheumatoid joint displays an activated prostaglandin E_2 _(PGE_2_) pathway, and there are high levels of this mediator in the synovial fluid and strong expression in the synovium of its synthesizing enzymes, microsomal prostaglandin E_2 _synthase 1 (mPGES-1) as well as cyclooxygenase (COX) 1 and 2 [1]. Wheres COX-1 is considered a constitutive enzyme present under basal conditions, COX-2 is inflammation-induced [2] and co-localizes with mPGES-1 in the synovial tissue [3]. The deleterious role of PGE_2 _in the pathogenesis of RA has already been established and occurs through multiple mechanisms. PGE_2 _sustains inflammatory pathways by promoting expansion of auto-aggressive T helper 17 (Th17) cells [4], increases angiogenesis within the proliferating synovium, and regulates cartilage and bone metabolism by stimulation of osteoclast and matrix metalloproteinase activity. Pro-inflammatory cytokines, such as tumor necrosis factor (TNF) and interleukin-1-beta (IL-1β), that orchestrate the pathological events in this disease are known inducers of mPGES-1 and COX-2 expression [5].

The levels of PGE_2 _are determined not only by its synthesis but also by the rate of degradation. Most of the prostaglandin inactivation occurs through the action of 15-hydroxyprostaglandin dehydrogenase (15-PGDH), which converts PGE_2 _to a metabolite with greatly reduced biological activity [6] and is thus the main mechanism for PGE_2 _clearance. 15-PGDH is ubiquitously expressed in most mammalian tissues [6]. In humans, IL-6 [7] and transforming growth factor-beta (TGFβ) regulate its expression in prostate and colon cancer, respectively, whereas TNF downmodulates it in colonocytes [8]. In addition, mediators controlling PGE_2 _formation reciprocally stimulate COX-2 while reducing 15-PGDH expression [9]. 15-PGDH decrease or loss was demonstrated in gastric [10], lung [11], and thyroid [12] tumors and results in increased malignant cell proliferation and cancer progression. In addition, reduced expression of 15-PGDH contributes to the elevated PGE_2 _levels observed in the systemic inflammatory response [13] or in the inflamed mucosa of patients with inflammatory bowel disease [8].

Recent reports demonstrated 15-PGDH synthesis in mouse articular chondrocytes and an inverse regulation of mPGES-1 and 15-PGDH by adipocyte-derived factors in these cells, resulting in boosted PGE_2 _levels [14]. Also, mechanical stress increases 15-PGDH mRNA expression in this system [15]. However, the presence and localization of this enzyme in human synovial tissue remain largely unknown.

Intra-articular glucocorticoids (GCs) are often used as efficient adjuvant therapy in RA to control for local inflammation. One of the mechanisms by which they achieve their anti-inflammatory effect relies also on inhibition of synovial mPGES-1 and COX-2 expression and formation of PGE_2 _[3]. Earlier reports evaluating the influence of dexamethasone on 15-PGDH demonstrated either induced expression in A549 cells [16] or inhibition of *in vitro *stimulated enzyme expression in a monocyte cell line [17].

In patients with RA, one of the most efficient drugs used in most cases as first-line therapy is methotrexate [18]. Its interference with the cellular folate metabolism results in immunosuppressive effects by inhibition of synovial inflammatory cell proliferation and enhancement of adenosine release. The influence of methotrexate on PGE_2 _production appears controversial; reports demonstrate inhibitory action in whole blood of patients with RA [19] and in fibroblast-like synoviocytes (FLSs) [20], but studies also point to no effect on PGE_2 _release by FLSs [21,22].

We undertook this study to investigate the distribution of 15-PGDH in synovial tissue and the effects of methotrexate therapy on the synovial expression of the PGE_2 _pathway enzymes in patients with newly diagnosed RA and *in vitro *in cultured FLSs. Moreover, we analyzed the change in expression pattern for 15-PGDH induced by local administration of GCs known to have potent anti-inflammatory properties.

## Materials and methods

### Patients and clinical data

Synovial biopsies were collected at the time of surgical joint replacement from eight patients with RA and five with ostheoarthritis (OA). In addition, synovial samples from three healthy individuals and three patients with psoriatic arthritis (PsA) were obtained by arthroscopy. The biopsies were used for detection and cellular localization of synovial 15-PGDH.

To investigate the effects of anti-rheumatic treatment on the enzymes of the PGE_2 _cascade, synovial biopsies were collected from two cohorts of patients. In the first group, 13 patients with newly diagnosed RA were enrolled; the median time period since diagnosis was 7 days, and the median time since the beginning of symptoms was 6.5 months. Table 1 illustrates the clinical and laboratory data of the patient cohort. These patients had active disease with a Disease Activity Score in 28 joints (DAS28) of greater than 3.2 (mean of 5.7). All patients fulfilled the American College of Rheumatology criteria for RA [23]. At baseline and 1 year after inclusion in the study, plane radiographs of the affected joint were evaluated for the presence of erosions. DAS28 was assessed at baseline and at a median of 3 months after treatment initiation. Patients were assigned to start methotrexate therapy with an initial dosage of 10 mg/week, which was increased in a stepwise manner to achieve the final dosage of 20 mg/week after 3 weeks. Synovial biopsies were obtained by arthroscopy from the same inflamed joint before methotrexate initiation and after a median of 8 weeks. Oral non-steroidal anti-inflammatory drugs (NSAIDs) and GCs in doses of up to 10 mg daily were allowed, as was a maximum of three intra-articular GC injections in joints other than the one biopsied.

**Table 1:** Demographic, clinical, and laboratory data of the methotrexate study cohort

Characteristics of cohort	Value
Number of patients	13
Median age (range), years	56 (32-78)
Number of males/females	4/9
Median interval from diagnosis (range), days	7 (1-25)
Median duration of symptoms (range), months	6.5 (2-12)
ACPA-positive patients, number (percentage)	6 (46)
Rheumatoid factor-positive patients, number (percentage)	9 (69)
Number of patients taking oral corticosteroids	2
Number of patients taking NSAIDs	10
Median duration between biopsies (range), days	58 (45-70)
Number of patients presenting erosions (percentage)	
At baseline	2 (15)
After 1 year	7 (53)
Mean DAS28 (minimum, maximum)	
At baseline	5.7 (3.9, 6.7)
After 3 months	3.8 (1.8, 5.8)

ACPA, anti-cyclic citrullinated peptide antibody; DAS28, Disease Activity Score in 28 joints; NSAID, non-steroidal anti-inflammatory drug.

In the second group, 10 patients who had RA and active knee arthritis were recruited to the study. The clinical features of this patient group were published previously [24]. All patients in the second group received an intra-articular injection of 40 mg of triamcinolone hexacetonide. Synovial biopsies were obtained prior to and a median of 10 days after treatment. All other anti-rheumatic medication was kept unchanged 2 weeks before the first arthroscopy and for the rest of the study period. All investigations were approved by the ethics committee of the Karolinska University Hospital, and participants gave written informed consent to participate in the study.

### Immunohistochemical evaluation

Frozen serial biopsy sections were fixed in 2% formaldehyde, and immunohistochemical staining was performed by using rabbit polyclonal anti-serum raised toward mPGES-1 [1], rabbit polyclonal anti-15-PGDH (Novus Biologicals, Littleton, CO, USA) and anti-COX-1 (Cayman Chemical Company, Ann Harbor, MI, USA), and mouse monoclonal anti-COX-2 (CX229; Cayman Chemical Company) antibodies in accordance with a published protocol [25]. Stained sections were quantitatively evaluated by computer-based image analysis, and results were expressed as the percentage of positive stained area versus total tissue staining. For immunofluorescence experiments, sections were incubated with a mixture of primary antibodies against 15-PGDH and cell markers for macrophages (CD163, clone Ber-MAC3), fibroblasts (prolyl-4-hydroxylase, clone 5B5), B cells (CD20, clone L26) (all from DakoCytomation, Glostrup, Denmark), T cells (CD3, SK7; BD Biosciences, San Jose, CA, USA), and endothelial cells (CD31, clone EN4; Novakemi, Handen, Sweden) followed by the addition of secondary antibodies conjugated to Alexa 546 or Alexa 483 (Invitrogen Corporation, Carlsbad, CA, USA).

### *In vitro *culture of fibroblast-like synoviocytes and measurement of prostaglandins

Commercially available FLSs (Dominion Pharmakine, S.L., Bizkaia, Spain) from patients with RA were grown in Dulbecco's modified Eagle's medium supplemented with 10% human serum and penicillin streptomycin (100 units/mL) in a humidified atmosphere containing 5% CO_2 _at 37°C. When reaching confluence, cells were passaged by gentle trypsinization and used between passages 4 and 6. The cells were incubated for 48 hours with 10 ng/mL IL-1β (R&D Systems, Abingdon, UK) in the presence or absence of 10 or 250 μM methotrexate. Unstimulated cells were used as controls. After treatment, supernatants were collected and frozen at -20°C until analyzed. Cells were trypsinised, washed twice in phosphate-buffered saline (PBS), and harvested for subsequent Western blot analysis.

Prostaglandin profiling of the primary prostaglandins (PGE_2_, PGD_2_, and PGF_2α_) and of the metabolites of PGI_2 _and tromboxane A_2 _(TxA_2_) - 6-keto PGF_1α _and TxB_2_, respectively - was accomplished by using liquid chromatography tandem mass spectrometry (LC-MS-MS). Cell culture supernatants were extracted with an Oasis HLB Extraction Plate (Waters Corporation, Milford, MA, USA) and analyzed by high-performance liquid chromatography by using Waters 2795 HPLC (Waters Corporation) coupled to a triple quadrupole mass spectrometer (Acquity TQ Detector; Waters Corporation). PGE_2_, PGD_2_, PGF_2α_, TxB_2_, 6-keto-PGF_1α_, and their corresponding deuterated standards were detected by using multiple reaction monitoring and quantified by using internal standard methodology and QuanLynx software (Waters Corporation).

### Western blot analysis of enzymes

Cells were harvested after the different treatments and protein was extracted by using cell lysis buffer (Pierce, Rockford, IL, USA) in accordance with the instructions of the manufacturer. Total protein concentration was determined by using the NanoDrop technique, and equal amounts of protein were separated on 4% to 12% NuPage polyacrylamyde gels (Invitrogen Ltd., Paisley, UK). The resolved proteins were blotted onto polyvinylidene fluoride membranes (Pall Life Sciences, Pensacola, FL, USA), which were further blocked in PBS-Tween-20 buffer containing 5% (wt/vol) non-fat dry milk for 1 hour at room temperature. Incubation with primary antibodies rabbit polyclonal anti-serum against mPGES-1, rabbit polyclonal anti-COX-2 (Cayman Chemical Company), rabbit polyclonal anti-15-PGDH (Cayman Chemical Company), or mouse monoclonal anti-β-actin (NeoMarkers, Freemont, CA, USA) was performed overnight at 4°C. After washing in 0.05% TTBS (Tris-Tween-buffered saline), the membranes were incubated with horseradish peroxidase-linked anti-rabbit IgG from donkey (GE Healthcare, Stockholm, Sweden), washed extensively in 0.1% TTBS, and finally visualized by a chemiluminiscence detection kit (Thermo Scientific, Rockford, IL, USA) on film (Amersham Hybond; GE Healthcare, Buckinghamshire, UK).

### Statistical analysis

For statistical analysis of the methotrexate and GC cohorts, we used the Wilcoxon test for paired samples, followed by Bonferroni corrections for multiple comparisons, whereas *in vitro *data were analyzed by the Mann-Whitney test for unpaired samples. *P *values of less than 0.05 were considered significant.

## Results

### Distribution of 15-PGDH in rheumatoid arthritis synovial tissue

**Figure 1:**
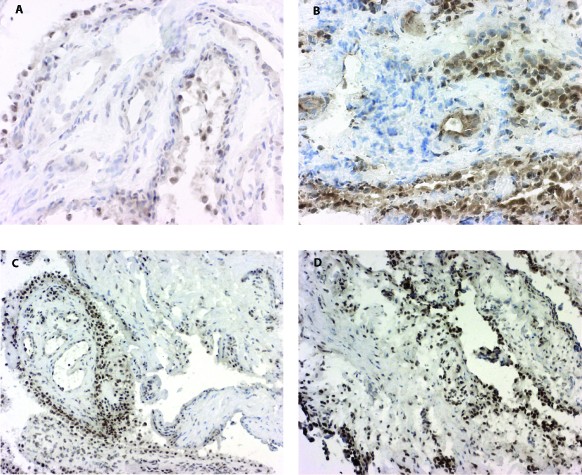
**Expression of 15-prostaglandin dehydrogenase (15-PGDH) in healthy and inflamed synovial tissue**. Immunohistochemical staining reveals positive (brown) staining for 15-PGDH (hematoxilin counterstained) in synovial tissue from healthy individuals **(a) **and patients with rheumatoid arthritis **(b)**, osteoarthritis **(c)**, or psoriatic arthritis **(d)**. Original magnifications: ×250 (a, b), ×100 (c, d).

All RA biopsy samples analyzed displayed 15-PGDH expression (Figure 1a). Its presence in RA synovial tissue was prominent in the synovial lining but also in scattered sublining macrophages and inflammatory infiltrates as well as vessels. Expression of 15-PGDH among patients was, nonetheless, variable; staining was strong and extensive in some patients and weak and confined mostly to the synovial membrane in others. In addition, the enzyme was present, albeit to a lesser extent, in healthy synovial tissue and in synovium from patients with OA and those with PsA (Figure 1b-d). Similarly to RA, the enzyme was found mainly in the lining layer in healthy tissue, PsA, and OA, although the expression was somewhat weaker in the lining synovial cells of healthy individuals compared with RA.

**Figure 2:**
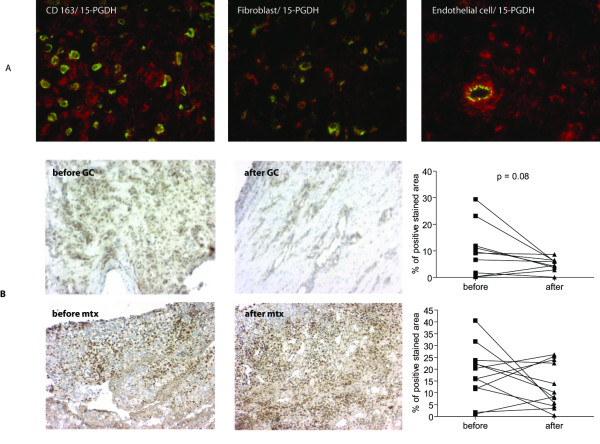
**Cellular localization of 15-prostaglandin dehydrogenase (15-PGDH) in rheumatoid arthritis synovial tissue and the effects of anti-rheumatic treatment on its expression**. **(a) **Double-immunofluorescence images show staining for 15-PGDH-positive (red) cells and cell marker staining (green) for macrophage CD163, fibroblast prolyl-4-hydroxylase, and endothelial cell CD31. Merged images display double-stained cells in yellow. Original magnification: ×400. Light microscopy pictures of representative synovial biopsy sections show immunohistochemical positive (brown) staining for 15-PGDH before and after intra-articular treatment with glucocorticoids **(b) **and before and 8 weeks after initiation of methotrexate therapy **(c) **(hematoxilin counterstained). Graphs display the comparative 15-PGDH expression in rheumatoid arthritis synovial tissue before and after treatment with glucocorticoids or methotrexate as a percentage of the positive stained area versus the total tissue area. GC, glucocorticoids; Mtx, methotrexate.

Most 15-PGDH-expressing cells were identified by double-immunofluorescence as CD163-positive synovial macrophages and fibroblasts in RA synovium (Figure 2a left and middle panels). In addition, most sublining vessels stained positively for 15-PGDH in endothelial cells (Figure 2 right panel) whereas CD20-positive B cells and CD3-positive T cells essentially lacked 15-PGDH enzyme (data not shown).

### Clinical evaluation and response to treatment

Out of the 13 patients included in the methotrexate study cohort, seven were responders, defined as those who had good or moderate response to treatment according to the European League Against Rheumatism (EULAR) criteria, and six were non-responders. The overall mean DAS28 decreased significantly to 3.8 after 3 months of treatment. All patients receiving intra-articular GCs were clinical responders as assessed by the local clinical examination of the joint and macroscopic evaluation of the local inflammation during the second arthroscopy.

### Effects of intra-articular steroid therapy on the expression of 15-PGDH in rheumatoid arthritis synovial tissue

We have earlier reported that intra-articular GC treatment downregulated the expression of PGE_2_-synthesizing enzymes in the rheumatoid synovium. Here, our results demonstrated that local GC therapy resulted in reduced expression of 15-PGDH in nine patients but that two patients showed increased values after therapy (Figure 2b). Overall, although no statistical difference (*P *= 0.08) was detected before or after local GC injection, there was a clear trend toward diminished enzyme expression following therapy.

### Effects of methotrexate therapy on the expression of 15-PGDH and related enzymes in rheumatoid arthritis joint

**Figure 3:**
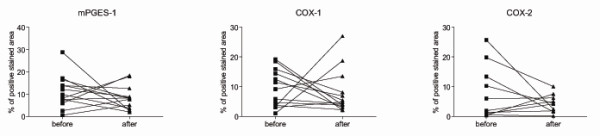
**Effects of methotrexate on the expression of prostaglandin E_2 _biosynthetic enzymes in rheumatoid synovium**. Immunohistochemical evaluation of positive staining for microsomal prostaglandin E_2 _synthase 1 (mPGES-1), cyclooxygenase 1 (COX-1), and COX-2 in synovial tissue biopsies before and 8 weeks after initiation of methotrexate treatment. Results are expressed as a percentage of the positive stained area versus the total tissue area.

We then studied the effect of oral methotrexate therapy on 15-PGDH and found that 8 weeks of oral methotrexate treatment did not significantly change the synovial expression level of 15-PGDH (Figure 2c). As the local PGE_2 _production is determined by its biosynthesis and degradation, we also examined whether methotrexate affects the biosynthetic enzymes. No effects of methotrexate on synovial expression of mPGES-1, COX-1, and COX-2 were detected (Figure 3). Stratifying patients according to response to treatment, presence of anti-cyclic citrullinated peptide antibodies, or erosion progression did not show any significant difference in enzyme expression between or within the groups (data not shown). We also found a similar overall staining pattern for PGE_2_-related enzymes irrespectively of NSAID treatment (data not shown).

**Figure 4:**
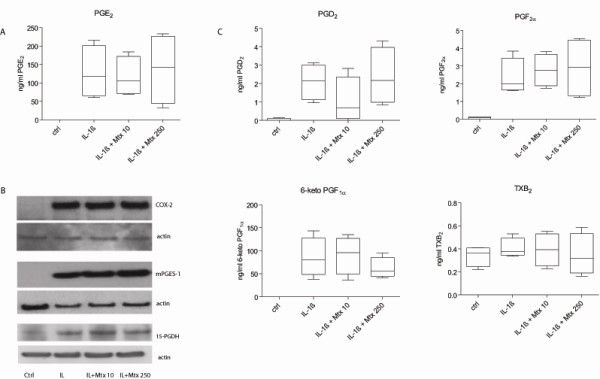
**Effects of methotrexate on the prostaglandin production by rheumatoid arthritis fibroblast-like synoviocytes (FLSs)**. Synovial fibroblasts were left untreated or activated with 10 ng/mL interleukin-1-beta (IL-1β) in the absence or presence of 10 or 250 μM methotrexate for 48 hours. After treatment, total protein was isolated and assayed for enzyme expression, while β-actin was used as loading control. Supernatants were analyzed for prostanoid content by using liquid chromatography tandem mass spectrometry. **(a) **Analysis of prostaglandin E_2 _(PGE_2_) formation in control and IL-1β-stimulated FLSs with or without methotrexate. **(b) **Representative Western blot results show expression of microsomal prostaglandin E_2 _synthase 1 (mPGES-1), cyclooxygenase 2 (COX-2), and 15-prostaglandin dehydrogenase (15-PGDH) in rheumatoid arthritis FLSs. **(c) **Analysis of PGD_2_, PGF_2α_, and the metabolites of PGI_2 _and TxA_2 _(6-keto PGF_1α _and TxB_2_, respectively) in culture supernatants indicates the amount formed after 48 hours of incubation. Horizontal lines indicate median values, and whiskers indicate range values (*n *= 4). Ctrl, control; Mtx, methotrexate; Tx, tromboxane.

In unstimulated *in vitro *cultured FLSs, low levels of PGE_2 _formation were mirrored by undetectable expression of COX-2 and mPGES-1 (Figure 4a, b). Still, FLSs displayed low 15-PGDH amounts even in basal conditions, and this is in line with the presence of this enzyme in most tissues. As expected, IL-1β determined a strong increase in PGE_2 _production along with upregulation of enzyme expression. The increase in 15-PGDH expression following IL-1β stimulation was, however, modest in comparison with the dominant upregulation of the synthesizing enzymes mPGES-1 and COX-2, explaining the net effect of increased PGE_2 _synthesis. However, the addition of methotrexate in either low or high concentration did not abrogate the IL-1β-induced effects on any studied enzyme expression or the PGE_2 _formation. Furthermore, analysis of the prostanoid profile by LC-MS-MS in supernatants from stimulated and unstimulated FLSs showed that methotrexate had no influence on the formation of any of the lipid mediators derived from the COX pathway, as demonstrated by detection of PGE_2_, PGD_2_, and PGF_2α _and metabolites of PGI_2 _or TxA_2 _(Figure 4c).

## Discussion

The contribution of the PGE_2 _pathway to the RA pathogenic process has been well established, and most studies have focused on PGE_2 _and its inflammation-dependent synthesizing enzymes. However, 15-PGDH, the enzyme responsible for the degradation of PGE_2_, has received little attention in this context, although its level of expression ultimately determines the availability of PGE_2_.

We described here for the first time the distribution pattern of 15-PGDH in synovial tissue in both pathological (inflammatory) and healthy conditions. 15-PGDH was present in patients with RA and was localized mostly in the lining macrophages and sublining fibroblasts and vessels. This finding is in agreement with localization of the mPGES-1 and COX enzymes in RA synovial tissue [1], indicating the possibility for local regulation and balance of formation and removal of PGE_2_. An alternative view is that, in an effort to overcome the high prostaglandin burden in RA, 15-PGDH expression increases as a protective mechanism. Furthermore, the enzyme was present in OA and PsA synovium, and this is in line with the association of these conditions with variable degrees of inflammation in synovial tissue. To a lesser extent, we also identified 15-PGDH in the synovial lining of healthy individuals, indicating constitutive formation of PGE_2 _in synovial tissue. Expression of PGE_2 _pathway enzymes has been reported in non-inflammatory conditions in kidney [26] and muscle [27], suggesting a possible role for basal PGE_2 _production in local tissue homeostasis.

Intra-articular GCs relieve local symptoms in the inflamed RA joint and are successfully used as add-on therapy to control for occasional bouts of inflammation. In this study, we demonstrated that GC injections may decrease the expression of 15-PGDH in synovial tissue that in turn, may promote local accumulation of PGE_2_. However, previous data from our group showed reduced expression of mPGES-1 and COX after local GC treatment [3], resulting in a diminished PGE_2 _generation. Thus, the attenuated 15-PGDH expression observed here may reflect simply a negative feedback induced by low PGE_2 _availability. Also, although PGE_2 _has a pivotal role in bone remodeling and degradation [28], complete removal of PGE_2 _may not be beneficial as there is evidence for a protective role for basal PGE_2 _in the resolution phase of inflammation [29-31] as well as for suppression of B-cell proliferation by PGE_2 _[32]. Furthermore, it is important to keep in mind that 15-PGDH, though the key enzyme for catabolism of prostaglandins, is also involved in degradation of lipoxins [33], with essentially opposed activity in the inflammatory milieu. As such, the lack of effect or trend toward decreased 15-PGDH reported here by GC may, in fact, contribute to preserving anti-inflammatory lipid mediators.

Given the limitation of the analytical system used in our study, we cannot rule out that the change in positive staining may be the result of a decrease in the number of inflammatory cells expressing the enzymes rather than an actual reduction in cellular expression. Data from a previous study demonstrated that intra-articular corticosteroid therapy reduced the number of synovial T lymphocytes but that the number of macrophages remained unchanged [34]. Since mPGES-1, 15-PGDH, and the COX enzymes are detected mainly in the macrophage and fibroblast populations but are essentially lacking in synovial lymphocytes, it is reasonable to assume that the changes we detect may be due to both reduced cellular enzyme formation and reduced inflammatory cell infiltration.

Despite being the first line of therapy for RA [35] and highly efficient in many patients, oral methotrexate showed no significant influence on the enzymes coordinating PGE_2 _metabolism. Image analysis of mPGES-1 expression, though not detecting any statistically significant change, showed that only four out of 13 patients displayed an increased mPGES-1 staining but that in all others methotrexate decreased the positive stained area. Thus, we cannot exclude an actual effect of the given therapy toward decreased mPGES-1, had we had a larger study cohort. On the other hand, the lack of effect on the 15-PGDH levels seen in our group of newly diagnosed subjects suggests local persistence of PGE_2 _in these patients. In fact, PGE_2 _availability despite methotrexate therapy could explain the progression in joint erosion seen in some of the responder patients. It is well known that methotrexate, through folate-dependent biosynthetic blockade, causes upstream accumulation of adenosine that turns on anti-inflammatory pathways by acting predominantly on A2A and A3 receptors [36]. In fact, experimental and clinical data suggest that the adenosine-mediated anti-inflammatory effect is the most prominent mechanism for low-dose methotrexate efficiency in RA. In this sense, inhibition of TNF [37] and IL-1β [38] actions and enhanced IL-10 [39] production were reported as indirect effects through adenosine release. Several reports suggest that methotrexate, administered either *in vivo *in animal models of arthritis [40] or added *in vitro *in different cell systems such as rat peritoneal macrophages [41] and human rheumatoid synoviocytes [20], may have inhibitory effects on PGE_2 _production. There is, however, evidence that methotrexate may fail to elicit a change in PGE_2 _in human fibroblasts [22,42]. Although study conditions and systems differ in the aforementioned studies, a clear and definite effect of methotrexate on the prostaglandin pathway in synovium-derived cells is not apparent. Our *in vitro *results demonstrated that, in RA FLSs, methotrexate had no influence on the PGE_2 _pathway or on any of the COX-derived lipid mediators. The increase in 15-PGDH expression following IL-1β treatment of synovial fibroblasts may be secondary to the high PGE_2 _amount formed under these circumstances, as PGE_2 _itself may induce 15-PGDH mRNA expression [43].

An earlier study evaluating synovial biopsies after 4 months of methotrexate treatment indicated that the macrophages and lymphocyte populations are reduced in the RA synovium [44]. Recent data from the same cohort of patients as the one used in our study demonstrated that 8 weeks of oral methotrexate therapy reduced the number of synovial CD3-positive lymphocytes and, to a lesser extent, the CD68-positive macrophages (Shankar Revu, submitted manuscript). Although lining and sublining macrophages highly express the enzymes involved in PGE_2 _formation, we found no significant influence exerted by methotrexate in our study. However, our study was designed to detect early changes in the synovium after the start of therapy and thus additional effects may become evident later on.

## Conclusions

15-PGDH is present in synovial tissue in conditions associated with inflammatory responses, such as OA, PsA, and RA, in a manner similar to that of the PGE_2_-synthesizing enzymes. Local GC treatment seems to reduce its expression, whereas oral methotrexate therapy exerts little influence in synovial tissue or in FLSs *in vitro*. Together, these results suggest that the inflammation-induced PGE_2 _pathway may not be properly targeted by anti-rheumatic treatment but instead persists and contributes to perpetuating inflammatory circuits in the rheumatoid synovium. As such, therapeutical attempts aiming at blocking the excessive production of PGE_2 _may be justified in order to offer additional benefit in reducing synovial inflammation and damage.

## Abbreviations

15-PGDH: 15-prostaglandin dehydrogenase; COX: cyclooxygenase; DAS28: Disease Activity Score in 28 joints; FLS: fibroblast-like synoviocyte; GC: glucocorticoid; IL: interleukin; LC-MS-MS: liquid chromatography tandem mass spectrometry; mPGES-1: microsomal prostaglandin E_2 _synthase 1; NSAID: non-steroidal anti-inflammatory drug; OA: ostheoarthritis; PBS: phosphate-buffered saline; PG: prostaglandin; PsA: psoriatic arthritis; RA: rheumatoid arthritis; TNF: tumor necrosis factor; TTBS: Tris-Tween-buffered saline; Tx: tromboxane.

## Competing interests

MK is employed part-time by Actar AB. The other authors declare that they have no competing interests.

## Authors' contributions

KRG participated in the study design, carried out the immunohistochemistry staining and analysis, performed statistical data analysis, and drafted the manuscript. SS carried out the Western blot analysis. IW performed the initial immunohistochemistry studies. PL participated in Western blot data collection and interpretation. HI carried out the mass spectrometry studies. AIC contributed to study design and collection of patient data and samples. MK conceived the study, participated in its design and coordination, and contributed to manuscript drafting and revision. P-JJ contributed to study design, data interpretation, and manuscript revision. All authors read and approved the final manuscript.
